# Seasonal and reproductive effects on wound healing in the flight membranes of captive big brown bats

**DOI:** 10.1242/bio.201410264

**Published:** 2014-12-19

**Authors:** Alejandra Ceballos-Vasquez, John R. Caldwell, Paul A. Faure

**Affiliations:** Department of Psychology, Neuroscience & Behaviour, McMaster University, Hamilton, ON L8S 4K1, Canada

**Keywords:** Cutaneous biopsy, Chiroptera, *Eptesicus fuscus*, Tissue regeneration and repair, Vespertilionidae

## Abstract

The flight membranes of bats serve a number of physiological functions important for survival. Although flight membrane injuries are commonly observed in wild-caught bats, in most cases the damage heals completely. Previous studies examining wound healing in the flight membranes of bats have not taken into consideration energy constraints that could influence healing times. Wound healing results in increased energy demands, therefore we hypothesized that wound healing times would be slower during periods of energy conservation and/or energy output. In this study we used an 8 mm diameter circular punch tool to biopsy the wing membranes of healthy adult female big brown bats (*Eptesicus fuscus*) from a captive research colony to test the hypothesis that healing times will vary with seasonal temperature changes between the summer and winter seasons, and with reproductive condition between lactating and non-reproductive females. As expected, membrane biopsies took significantly longer to heal during the winter when bats were hibernating compared to the summer when bats were active. Surprisingly, no difference in healing time was observed between lactating and non-reproductive females. The wings of most bats fully healed, although some individuals showed wound expansion demonstrating that impaired healing is occasionally observed in otherwise healthy subjects.

## INTRODUCTION

Bats (Order Chiroptera) are the only mammals capable of powered flight. In addition to providing lift and powered locomotion, the flight membranes or patagia of bats play a vital role in thermoregulation and homeostasis. The patagia are comprised of skin extending between the trunk of the body and the fifth digit (plagiopatagium), between digits V and II (chiropatagium), and many species have a tail membrane extending between the hind limbs (uropatagium). The patagia comprise ca. 85% of the total body surface area of a bat (Makanya and Mortola, 2007). While superficially similar to other types of mammalian skin, the morphology and thickness of the flight membranes of bat typically differs from other mammals and even from the skin of other body parts in bats (Crowley and Hall, 1994; Gupta, 1967).

Bat flight membranes are composed of a dorsal and ventral layer of epidermis separated by a central region of connective tissue consisting of collagen and elastin bundles (Gupta, 1967; Holbrook and Odland, 1978; Murphy, 1960). There is contention over whether the patagia contain dermal and hypodermal layers. A recent histological study of the wing of the common pipistrelle (*Pipstrellus pipistrellus*) divides the patagium into two components: a scaffold area where dermal and hypodermal layers are observed, and a thin area where these layers are not well defined (Madej et al., 2012). Despite variation in the composition of the wing, a consistent feature is that this membrane is significantly thinner than skin covering the rest of the body (Gupta, 1967; Madej et al., 2012) making it prone to holes, tears, and other injuries.

Like other types of skin, the patagia act as a barrier and are important in a number of physiological functions. For example, the wing membranes play a role in cutaneous gas exchange (Makanya and Mortola, 2007), thermoregulation (Kluger and Heath, 1970), and water balance (Cryan et al., 2010; Willis et al., 2011). Given their importance in locomotion and homeostasis, the flight membranes of bats have likely been under strong natural selection to heal quickly.

Despite the prevalence of flight membrane injuries in the wild (Davis, 1968), there have been few experimental studies on wound healing in bats (see Church and Warren, 1968; Davis and Doster, 1972; Iversen et al., 1974; Faure et al., 2009; Weaver et al., 2009). Presumably, the process of regenerating flight membrane tissue requires bats to mobilize and/or re-allocate energy resources, yet nothing is known about membrane healing during times of energy constraint or peak energy demand. Because biologists routinely punch bat flight membranes to collect tissue for molecular analyses, genetic studies, and/or to temporarily mark animals in the field, a better understanding of the factors that influence wound healing is warranted.

Wound healing is an immunological response to an injury that disrupts normal tissue homeostasis. In mammals, wound healing is a complex process involving highly organized sets of events regulated by different genes, cytokines, and hormones that are turned on and off at specific times (Singer and Clark, 1999; Gurtner et al., 2008). Owing to this complexity, wound healing is thought to require a substantial energy investment (Ennis et al., 2007; Im and Hoopes, 1970). Indeed, wound healing has even been as proxy for studying energy allocation and immune competence (French et al., 2007; Rees et al., 2001). The healing process consists of an overlapping series of actions that occur in four phases – hemostasis, inflammation, cell proliferation, and remodelling (Guo and Dipietro, 2010) – subdivided according to cellular and physiological events. Hemostasis occurs immediately after tissue injury and consists of vasoconstriction and clotting. In the second phase, an inflammatory response is mounted and neutrophils, macrophages, and lymphocytes are recruited to the injury site. During cell proliferation extensive extensive re-epithelialization, angiogenesis, and collagen synthesis occurs. During the final remodeling phase, which overlaps with cell proliferation and can take years to complete, there is collagen remodelling and blood vessels formed during angiogenesis regress. Our study focused mainly on the inflammation and proliferative phases because both are easy to observe and measure as new tissue forms and closes the wound.

Because wound healing results in increased energy demands (Im and Hoopes, 1970), it has been used as proxy for studying energy allocation and immune competence (French et al., 2007; Rees et al., 2001). In this study, we hypothesized that flight membrane healing times in the big brown bat (*Eptesicus fuscus*) would vary across seasons and during times of increased energy expenditure. We predicted that wound healing would be slower during periods of energy conservation (e.g. hibernation) and during peak energy demand (e.g. pregnancy and lactation; Barclay, 1989; Barclay, 1991). Thus, we expected wound healing would be slower during the winter compared to the summer, and also for lactating compared to non-reproductive females. In line with the latter prediction, we also measured pup growth to evaluate whether a trade-off exists in energy allocation between healing and pup growth.

To test these hypotheses, we inflicted wounds in the flight membranes of captive female *E. fuscus*. We were particularly interested in wing wound healing in the winter because it is during hibernation when bats experience the most devastating effects of infection with the fungus *Pseudogymnoascus destructans*, the causative agent of white nose syndrome (WNS) that has killed millions of bats in North America. It has been suggested that bats harboring *P. desctructans* are compromised in their ability to regulate water balance and are susceptible to dehydration owing to increased evaporative water loss (EWL) across their damaged flight membranes, resulting in infected bats arousing more frequently to drink during the winter and thus using up their fat stores (Cryan et al., 2010; Willis et al., 2011; Warnecke et al., 2013). The “dehydration hypothesis” predicts that bats with wing wounds compensate for this increased water loss by increasing water consumption. To test this, we monitored bat drinking behaviour during the winter to determine whether biopsied bats compensated for increased evaporative water loss by having a higher drinking frequency compared to non-biopsied control bats living with them in the colony. To the best of our knowledge, this is the first study to examine the influence of seasonal temperature and energy expenditure on flight membrane healing times in bats. The results of this study will provide bat biologists with important baseline healing information relevant for animals that have survived an infection with WNS.

## RESULTS

### Gross morphological observations

**Fig. 1.**
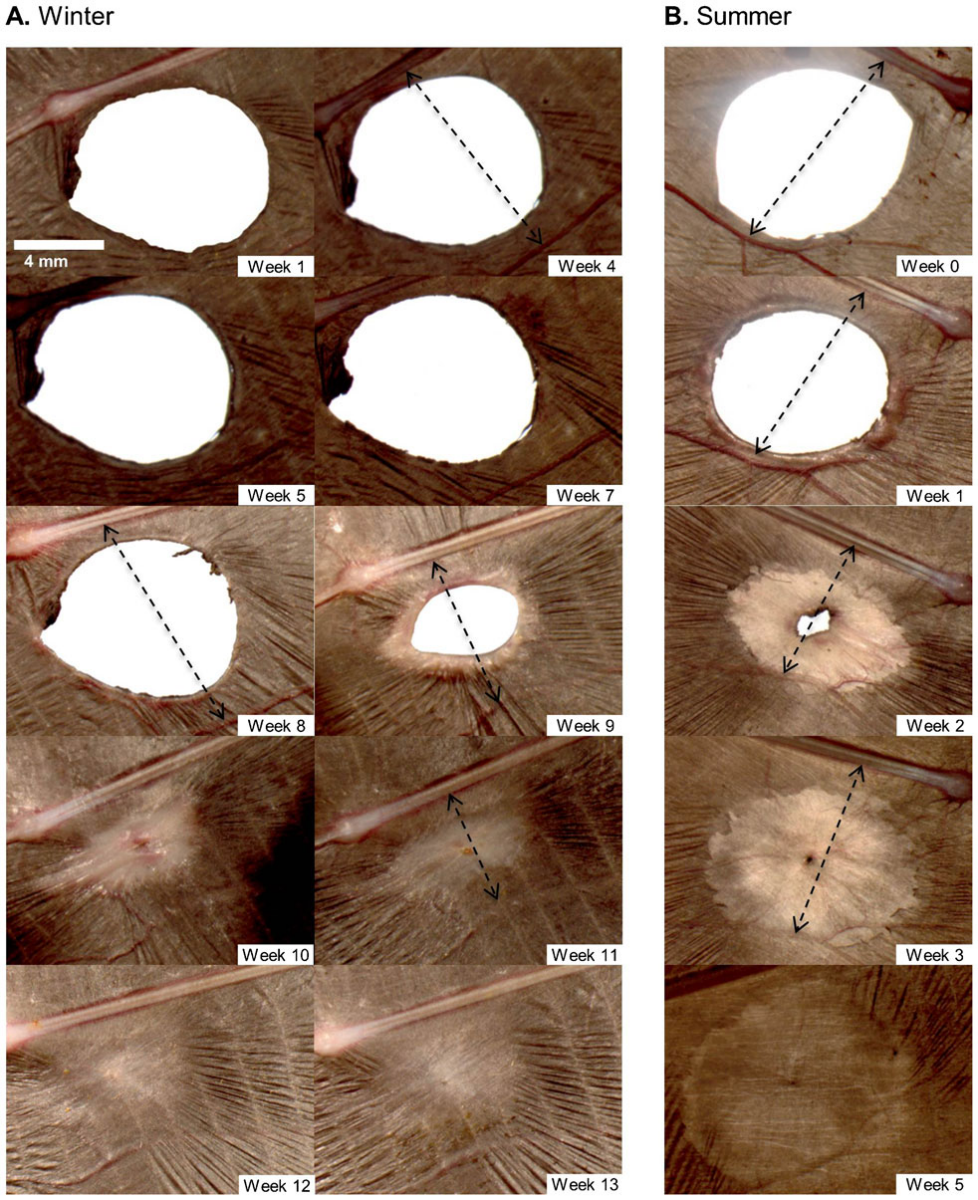
Progression of wing wound healing during the winter and summer by captive adult female *E. fuscus* in the weeks following biopsy. (A) Right wing of Female B02 from winter treatment group. (B) Left wing of Female B22 from summer treatment group. Scale bar in panel A (Week 1) applies to all images. Arrows indicate tissue contracture around the wound (measurements given in text).

**Fig. 2.**
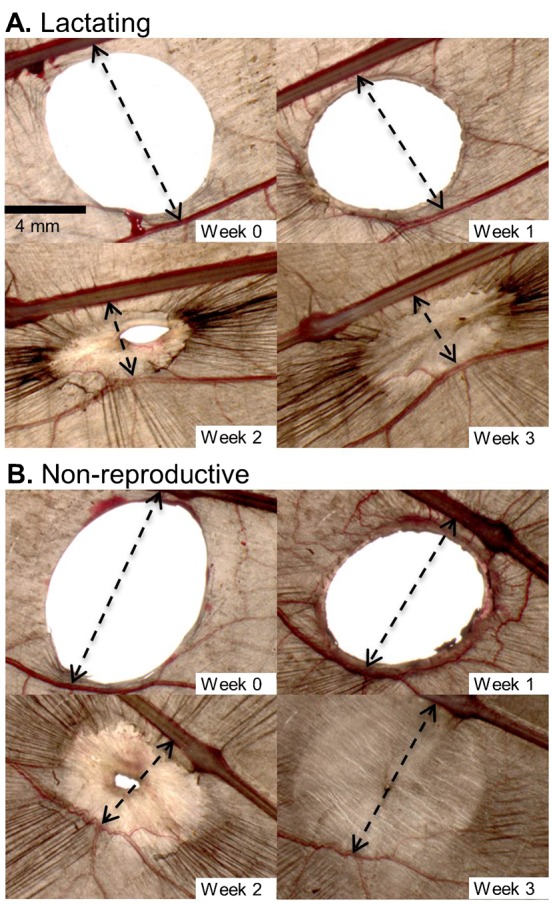
Progression of wing wound healing for lactating and non-lactating captive adult female *E. fuscus* in the weeks following biopsy. (A) Right wing of P44 from lactation treatment group. (B) Left wing of P38 from non-reproductive treatment group. *Scale bar* in panel A (Week 0) applies to all images. Arrows indicate tissue contracture around the wound (measurements given in text).

During the winter, the appearance of the wing was largely unchanged and there was no clotting, scabbing, or inflammation of the wound during the first 5 weeks after biopsy (Fig. 1A). By week 6, inflammation started to develop around the margins of the wound and the surrounding vasculature became more pronounced. Once the wound began to heal, the morphological changes to the wound were quite similar in the winter/summer and lactating/non-reproductive treatment groups (Figs 1, 2), although wound inflammation in the winter group seemed less pronounced compared to the other groups. Re-epithelialization was observed as the wound area decreased in size. The newly formed tissue was noticeably thinner and paler than the surrounding tissue that was not biopsied. As new tissue was added a reduction in surface area (i.e. a contracture) was observed in the tissue closest to the biopsy location in all healing bats from every treatment group (see arrowheads in Figs 1, 2). To illustrate the contracture, we measured the change in linear distance between the 4^th^ digit and a major blood vessel running posterior to the wound in one animal from every treatment group. The bone-to-vessel distance in Blu002 (winter) was 9.84 mm in week 4, 9.85 mm in week 8, 6.86 mm in week 9, and 5.52 mm in week 11. In Blu022 (summer) the initial bone-to-vessel distance was 10.14 mm in week 0, but this decreased to 8.98 mm by week 1, and 6.10 mm by week 2, but then increased to 8.04 mm at week 3. Female P44 (lactating) had an initial bone-to-vessel distance of 9.78 mm in week 0, that was 8.06 mm by week 1, 4.05 mm by week 2, and 4.17 mm by week 3. Finally, bat P38 (non-lactating) had an initial bone-to-vessel distance of 10.47 mm in week 0, that decreased to 9.10 mm by week 1, was 5.53 mm by week 2 but increased to 7.86 mm by week 3.

During the healing process the edges of the wound thickened and in some individuals became red and inflamed. In some individuals we observed the formation of new blood vessels in the wing tissue surrounding the wound (e.g. Fig. 1B, Fig. 2AB: weeks 1 and 2). Wounds that were nearly healed often had a small pin-hole in the center of the scab covering the wound (e.g. Fig. 1A, week 11). In some cases when this scab fell off there was incomplete wound closure.

Once the wound had closed and the process of inflammation and cell proliferation had ceased, the remodelling phase commenced. As newly formed tissue slowly acquired pigment it becomes less distinguishable from the surrounding tissue (e.g. Figs 1, 2); however, because wrinkles in the chiropatagium had not reformed, thus newly healed tissue remained smooth and distinct compared to uninjured tissue (e.g. Fig. 1A, week 13). The smoothness of the newly formed tissue was observed in all bats and was evident even after one year post-biopsy.

**Fig. 3.**
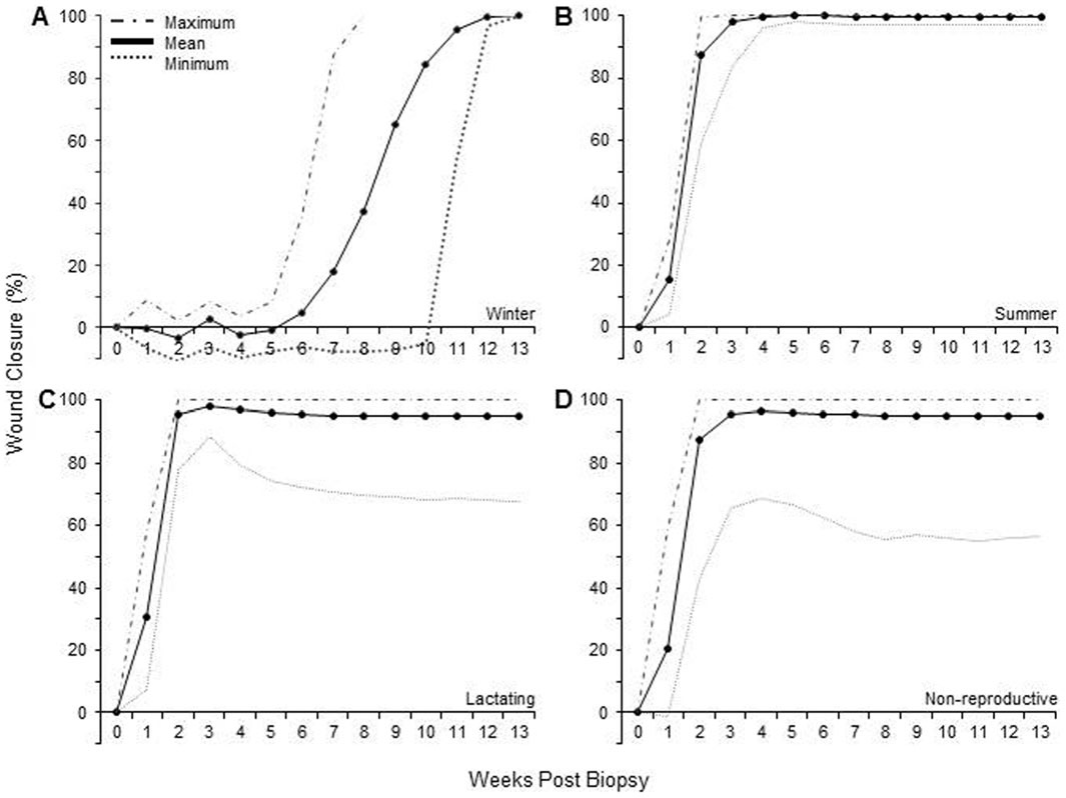
Wound healing as a function of the number of weeks post-biopsy in captive adult female *E. fuscus*. Each panel shows the mean (*solid line*), maximum (*dashed line*), and minimum (*dotted line*) percent wound closure calculated as a normalized area (see Materials and Methods); negative values indicate wounds that increased in size re Week 0. (A) Winter treatment group, n = 12. (B) Summer treatment group, n = 14. (C) Lactating treatment group, n = 20. (D) Non-lactating treatment group, n = 16. Legend in A applies to all panels.

**Fig. 4.**
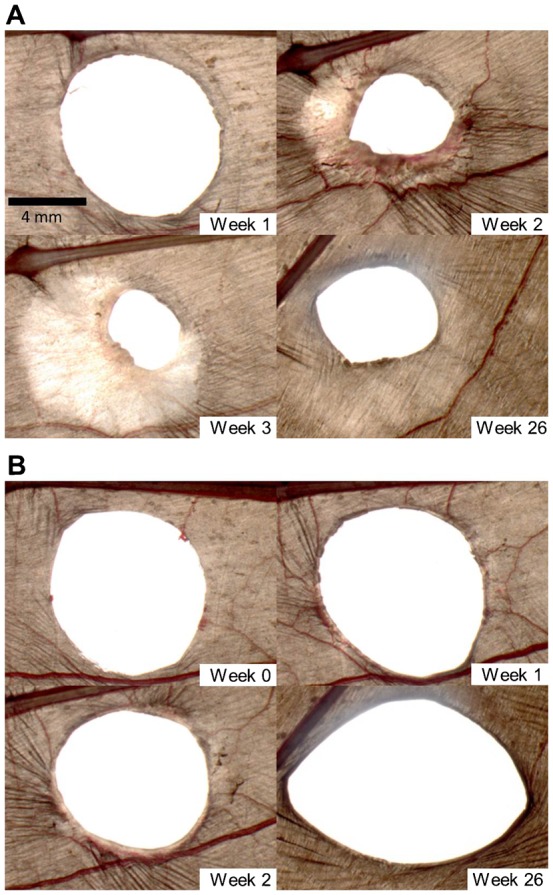
Examples of impaired wound healing with wound expansion in captive female *E. fuscus* in the weeks following biopsy. (A) Right wing of lactating female P08. (B) Right wing of lactating female B41. Scale bar in panel A (Week 1) applies to all images.

Most bats biopsied in the summer, when temperatures were warmer, reached full wound closure within 3 to 5 weeks (Fig. 3B–D). Wounds that had not fully closed by this time followed the same process of healing during their initial 3 to 5 weeks, but subsequent to this we observed no signs of epithelial proliferation and/or a decrease in wound area. The wound area in some individuals increased after week 4 (Fig. 3C,D). In these cases there was no sign of inflammation or swelling around the wound edges, and the blood vessels surrounding the wound were less prominent. In this paper we report on wound healing for up to 13 weeks following biopsy, although we continued to monitor 14 of 15 bats that had not healed by 26 weeks post-biopsy; in 10 cases there was very little or no change to the wound area after week 6, and in 4 cases the wound area expanded. Twelve of these 14 bats were re-photographed ca. one year later and in 3 cases there was no change in the area of the persisting wound. Of the remaining 9 bats, 8 had wound areas larger than last measured on week 26, and 1 showed a decrease in wound area. In all 14 bats the tissue surrounding the wound appeared to be in the remodelling phase of healing (e.g. compare Fig. 4A, week 3 with week 26).

Although not directly related to our study, it is noteworthy that during late winter and early spring we observed depigmentation in the flight membranes of all bats. The degree of wing paleness and translucency varied greatly across individuals, with some showing little depigmentation while others presented wings that were close to being uniformly unpigmented.

### Part I. Effect of season on wound healing

**Fig. 5.**
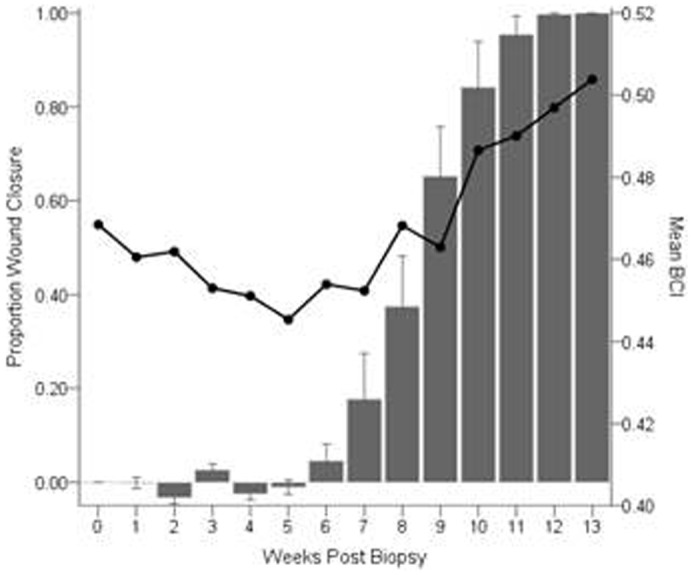
Mean ± SE proportion wound closure and mean Body Condition Index as a function of weeks post biopsy in captive adult female *E. fuscus*. Normalized proportion wound closure (*solid bars*, left y-axis; n = −12) calculated as ([initial area-current area]/initial area). Bats were biopsied on week 0 (17 January 2013), and all animals were fully healed by week 13 (18 April 2013). Mean BCI (*solid line*; right axis) for the same group of bats.

There was no difference in body condition index (BCI) score between the winter biopsy (experimental) and no biopsy (control) treatment groups (Wald 
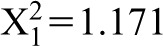
, p>0.05). Bats in both groups experienced a decrease in body mass between week 0 and week 5, but started to gain weight on or after week 6. The increase in body mass was mirrored by food consumption; bats in both groups ate on average of 0.9 g/day/bat between weeks 0 and 5, which increased to 3.7 g/day/bat between weeks 6 and 13. Wound healing was not observed in the winter biopsy group during the initial 5 weeks, but was evident thereafter once the bats became more active and started gaining mass (Fig. 5). The increase in activity was inferred from an increase in motion triggered files collected. There was no difference in the frequency of drinking between the biopsy and non-biopsy treatments during the winter (Pearson Chi-square test: X^2^ = 0.6929, d.f. = 2, p>0.05).

In contrast to the winter, wound healing was observed within the first week following wing membrane biopsy in the summer (see Fig. 1B). Complete wound closure occurred for some bats by week 3, and 13 of 14 bats (92.8%) had fully healed by week 5. On average full wound closure (once signs of wound healing were observed) during the summer was achieved in 4 weeks, while the same average for the winter group was 7 weeks (see Fig. 3).

**Fig. 6.**
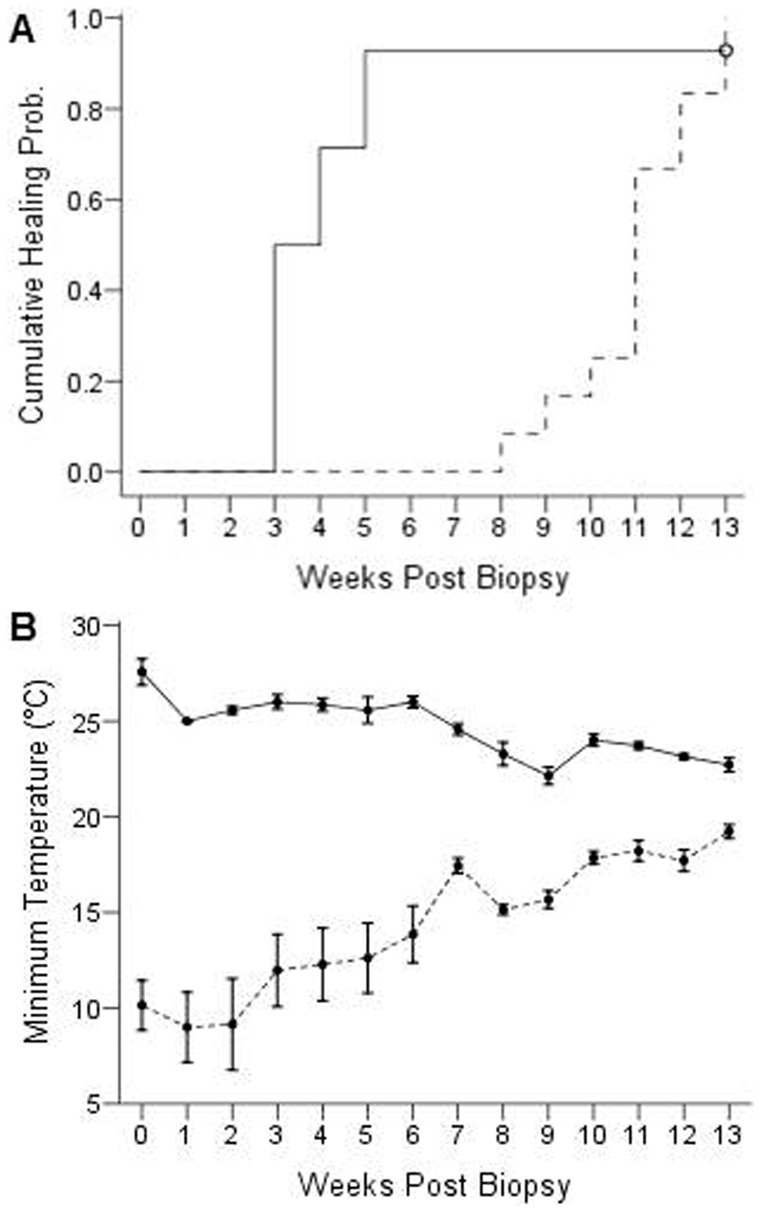
Probability of healing and minimum colony temperature for the winter and summer treatment groups as a function of weeks post biopsy in captive adult female *E. fuscus*. (A) Time-to-event analysis for wing wound closure in the winter (*dotted line*; n = 12) and summer (*solid line*; n = 14) seasons. There was a significant effect of season on healing times. (B) Mean (± SE) weekly minimum colony temperature in the bat husbandry facility in the winter (*dotted line*; 17 January to 18 April 2013) and summer (*solid line*; 18 July to 10 October 2013).

There was a significant effect of season on wound healing times at the beginning (Mantel-Cox = 9.16, p = 0.002), during the middle (Generalized Wilcoxon = 15.85, p<0.0001), and at the end of the study period (Tarone-Ware = 13.99, p<0.0001). Weekly minimum temperatures differed greatly between the winter and summer seasons in the husbandry facility where the bats were housed (Fig. 6B). The mean ± standard error (SE) minimum weekly temperature during the winter portion of the study was 14.3 ± 0.5°C. During the first 5 weeks following membrane biopsy and before healing was observed, the average minimum temperature was 10.8 ± 0.7°C, but increased to 16.9 ± 0.3°C between weeks 6 through 13 when healing was in progress. The average minimum temperature during the weeks when healing was observed in the summer study was 24.6 ± 0.2°C.

### Part II. Effect of reproductive status on wound healing

**Fig. 7.**
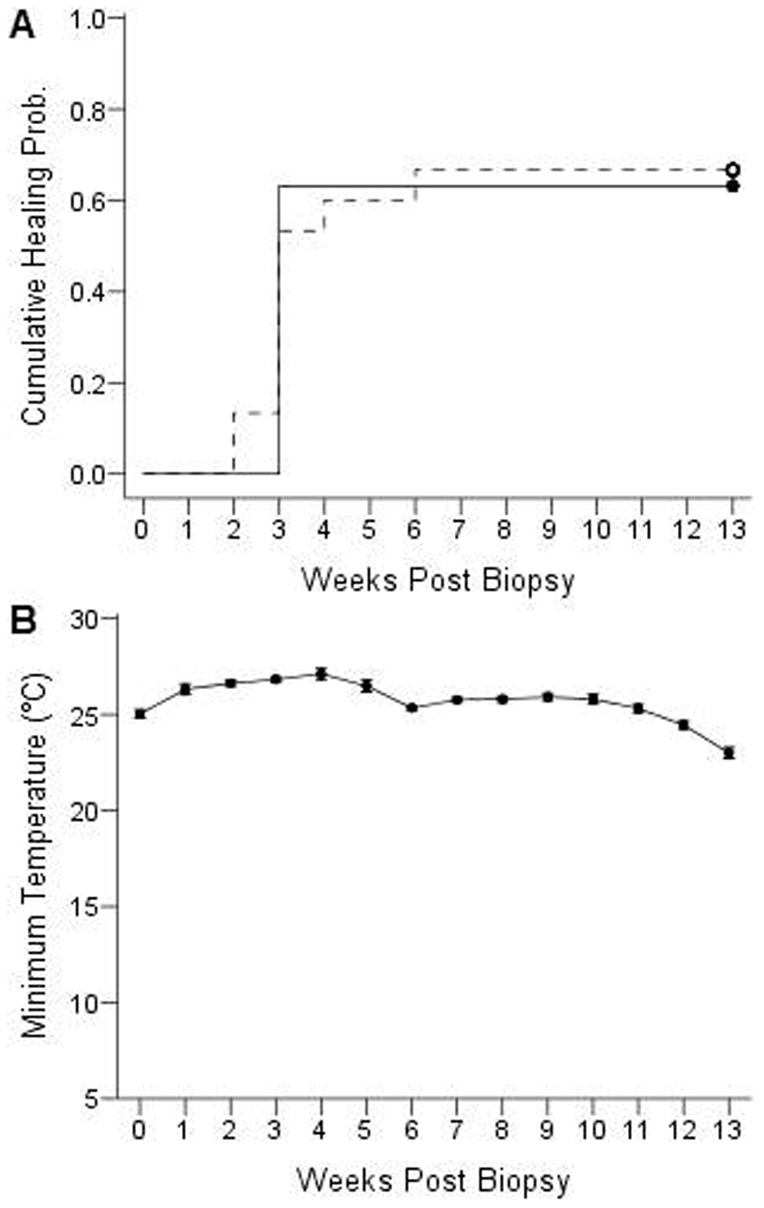
Probability of healing and minimum colony temperature for the lactating and non-reproductive treatment groups as a function of weeks post biopsy in captive adult female *E. fuscus*. (A) Time-to-event analysis for wing wound closure in the lactating (*dotted line*; n = 20) and non-reproductive (*solid line*; n = 16) females. There was no effect of reproductive conduction on healing times. (B) Mean (± SE) weekly minimum colony temperature in the bat husbandry facility during the experiment (10 June to 19 October 2013).

Surprisingly, there was no difference in healing time between lactating and non-reproductive adult females (Fig. 7A) at the beginning (Mantel-Cox = 0.24, p = 0.62), during the middle (Generalized Wilcoxon = 15.85, p = 0.84), or at the end of the study period (Tarone-Ware = 0.98, p = 0.75). Weekly minimum colony temperatures were fairly consistent between weeks 0 and week 5 when wound healing was most rapid (Fig. 7B). Moreover, the mother's healing time (Linear mixed-effects model, p = 0.3780) and whether she reared one or two offspring (Linear mixed-effects model, p = 0.7904) had no influence on pup growth rate; the average rate of mass gain was 2.58 ± 0.08 g and the average rate of forearm length growth was 5.01 ± 0.13.

## DISCUSSION

Given that tissue regeneration and repair is an energetic process, we hypothesized that wound healing would be slower during periods of peak energy conservation (i.e. hibernation) and/or demand (i.e. lactation). Hence, we predicted that wound healing in *E. fuscus* would be slower in the winter *versus* the summer, and for lactating *versus* non-reproductive adult females. The results from our summer *vs.* winter healing experiment supported this prediction, with hibernating *E. fuscus* experiencing delayed wound healing compared to active bats in the summer. Contrary to our prediction, there was no difference in healing time between lactating and non-reproductive adult females. Unexpectedly, *E. fuscus* nursing young were able to successfully partition energy into wound repair and reproduction and their wound healing was not delayed compared to non-reproductive females.

### Seasonal effect on wound healing

Whereas the winter treatment group did not show healing until week 5, the summer group showed healing within 1 week following biopsy. Our gross morphological observations suggest that wound healing during the winter was mainly delayed rather than slowed, a finding that disagrees with previous suggestions that wound healing should be slower during hibernation (e.g. Wilmer and Barratt 1996; Weaver et al., 2009). The finding of delayed wound healing during winter hibernation is contrary to observations of winter wound healing in other hibernating mammals. For example, American black bear (*Ursus americanus*) fully healed skin biopsies during the winter and healing progressed normally throughout all of its phases (Iaizzo et al., 2012). Perhaps the difference in winter wound healing between *E. fuscus* and *U. americanus* can be accounted for by different body temperatures that are maintained during hibernation; hibernating bats allow their core body temperature to vary with ambient while hibernating bears maintain their body temperature well above ambient (i.e. 31–35°C; Nelson et al., 1983).

Wound healing is an immunological response to injury and tissue disruption, and has been proposed as an integrative measure of immune function (Archie, 2013). A number of mammals experience immunological suppression during the winter (for a review, see Bouma et al., 2010). If the bats in our study were immunologically suppressed during hibernation, then it may not be surprising that their wound healing was delayed (Fig. 3). Although we attribute slower healing to winter hibernation, it is important to note that delaying healing most likely resulted because of lower ambient temperatures and/or reduced food consumption by the bats. The time of arrested healing occurred during weeks 0–5, and this coincided with the period of coldest colony temperatures, reduced food intake, and decreased body mass in our bats. Wound healing in hibernating bats occurred mainly after week 6, which coincided with a period of warmer colony, temperatures, higher daily activity, and increased food consumption. In contrast to our expectations, wound healing by hibernating bats did not result in faster depletion of fat reserves because there was no difference in BCI between bats in the winter biopsy and winter control groups. The role of nutrition in wound healing is still a matter of controversy. Some studies indicate caloric restriction improves wound healing, while others suggest the opposite (see Albina 1994).

In contrast to the dehydration hypothesis (e.g. Cryan et al., 2013; Warnecke et al., 2013; Willis et al., 2011), there was no difference in the frequency of drinking behaviour between the wing biopsy and control treatment groups during winter hibernation. This result suggests that bats with biopsied wing membranes did not experience greater evaporative water and did not need to arouse more frequently to drink during hibernation; however, we did not measure water consumption so we cannot exclude the possibility that bats with damaged wings consumed more water per drinking bout rather than by increasing their drinking frequency. Future studies are needed to test these possibilities.

Wound healing during the summer progressed rapidly. Except for one female that did not heal, all bats in the summer treatment group reached 100% wound closure in both wings by week 5. Our finding is consistent with the study by Davis and Doster (Davis and Doster, 1972) who reported that 14-mm and 17-mm diameter circular holes in the wings of *A. pallidus* healed by 34 and 43 days, respectively. In a separate study, Faure et al. (Faure et al., 2009) reported that 8-mm diameter circular holes in the wings of *E. fuscus* required, on average, 127 days to heal. Although the biopsy size and location in the present study were identical to that of Part II in Faure et al. (Faure et al., 2009), a difference in healing time may have resulted because of animal handling and seasonal influences. The bats in Faure et al. (Faure et al., 2009) were handled and photographed every second day, whereas in the current study bats were measured weekly. The study by Faure et al. (Faure et al., 2009) was conducted between September and March, and although they used a heating fan to maintaining the colony temperature during the winter (mean temperature = 21.5°C), perhaps the use of artificially heated and blowing air influenced healing times. The difference in healing time between this study and Part II of Faure et al. (Faure et al., 2009) supports the conclusion that there are strong temperature (and possibly other seasonal) effects.

### Reproductive effects on wound healing

Contrary to our prediction, there was no difference in healing time between lactating and non-reproductive female *E. fuscus*. Wound healing progressed rather quickly and females in both groups had fully healed by week 3. Our results clearly demonstrate that bats in captive with *ad libitum* access to food can rapidly heal their wings even when faced with the energetic demands of nursing their pup(s). Weaver et al. (Weaver et al., 2009) and Dobony et al. (Dobony et al., 2011) reported field observations of wound healing in lactating female bats, although neither described the fate of their offspring. While it is certainly possible that lactating females in the field would have less energy reserves available to allocate to milk production (i.e. the rearing of their pups) and wound healing, the lactating females in our captive study healed completely and successfully reared either one or two offspring. One must be cautious in extrapolating this result to bat populations in the wild because the bats in our study were not food limited and could during the day and night. Female bats in our study could have compensated for the higher energy demands associated with lactation and tissue repair by increasing their food intake. McLean and Speakman (McLean and Speakman, 1999) suggested that brown long-eared bats (*Plecotus auritus*) compensate for the expenditure of lactation by increasing food consumption. A trade-off between reproduction and healing has been documented in breeding tree lizards (*Urosaurus ornatus*) forego wound healing when food is limited (French et al., 2007). Future studies controlling for food intake are needed to determine if such a trade-off exists in insectivorous bats when resources are limited.

### Implications of wound healing in bats

Although the patagia of bats serve both locomotory and physiological roles, the importance of flight membrane integrity on these functions is unclear. During healing the skin surrounding the wounds contracts, resulting in a reduction in the flight membrane surface area between the bone of the 4^th^ digit and the first major blood vessel below the wound (Figs 1, 2), but whether wound contraction influences locomotion remains an open question. We know bats can fly with very large holes in their wings (e.g. Davis, 1968), but we don't know if the wounds influence flight performance. Billingham and Russell (Billingham and Russell, 1956) discussed movement limitations in human caused by healing skin, so it seems possible that wound contractures may limit movements and/or cause flight impairments in bats because their wings are delicate organs highly innervated with somatosensory receptors (Chadha et al., 2011; Sterbing-D'Angelo et al., 2011). Reichard and Kunz (Reichard and Kunz, 2009) suggested that membrane wounds negatively affect the physiology of bat wings, thereby limiting flight performance decreasing foraging success. Voigt (Voigt, 2013) studied flight performance in two species of wild *Myotis* with wing membrane defects and reported that injured animals performed fewer U-turns, suggesting flight membrane wounds have functional consequences by impacting the ability of bats to forage and evade aerial predators. Bats with severe wing damage following an infection with the fungus *P. destructans* have lower BCI scores, but it is unclear if their ability to forage was compromised by the damage to their flight membranes (Fuller et al., 2011).

We noticed that newly healed wing tissue was smooth and did not take on the wrinkled appearance that is common to older tissue surrounding a wound site. The wrinkling is caused by microscopic collagen and elastin bundles located within the flight membrane (Holbrook and Odland, 1978). That newly formed tissue in the wing does not have the same wrinkled morphology as uninjured wing tissue clearly demonstrates that flight membrane injuries do not result in full tissue repair, as been previously suggested (van Bekkum, 2004; Goss, 1987; Maginnis, 2006). At present, the functional consequences for a bat with smooth *versus* wrinkled wing membranes are unknown.

### Impaired wound healing

Most of the wounds we inflicted healed completely within 13 weeks, but some did not fully close or heal at all (e.g. Fig. 4). In Part I of our study, one female in the summer treatment group had a wound that was 97% closed before healing arrested. In Part II of our study, impaired healing was observed in 8 of 20 (40%) experimental and 6 of 16 (37%) control bats – a difference that was not significant. Although we continued to monitor bats that had not fully healed for up to 26 weeks, the wounds of these individuals remained largely unchanged. We have also observed impaired wound healing while conducting neurophysiological studies on bats but have been unable to attribute a cause for the impairment. Impaired wound healing was reported by Dobony et al. (Dobony et al., 2011), who noted that some recaptured lactating female *Myotis lucifugus* had wing wounds that had not healed. Although Dobony et al. (Dobony et al., 2011) attributed the impaired healing to WNS, suggesting the bats were unable to allocate sufficient energy to both reproduction and wound repair, this cannot explain the impaired healing observed in our non-reproductive, captive female *E. fuscus* because they were not infected with *P. destructans*. Moreover, wing damage has been noted in bats prior to the emergence of WNS (Powers et al., 2013). Given that impaired healing seems to be a natural phenomenon in healthy bats, including both lactating and non-reproductive females, we caution researchers to avoid concluding that impaired healing in wild bats is a result of a previous WNS infection.

Our observations on bats with wounds that had not fully healed by week 6 indicates that healing continued to progress through each stage even though the wound area may have been unchanged for up to 51 weeks. Perhaps these bats progressed to the re-epithelialization stage of healing but then arrested before wound closure was achieved. Many bats healed to the point where only a pin-sized hole was present in the wing, and in a few cases this hole expanded over time. Although pin-holes are unlikely to cause adverse effects, because they have the potential to enlarge they could compromise the integrity of the wing. Fluorescence imaging on the wings of *Myotis* spp. have shown that even minute needle stabs activate the lymphatic system (Murphy, 1960), thus it seems prudent not to underestimate the physiological effects of small holes in the flight membranes.

With the emergence of WNS, there has been renewed interest in understanding membrane healing in bats. Fuller et al. (Fuller et al., 2011) and Dobony et al. (Dobony et al., 2011) reported that bats had more severe wing damage early in the summer, but later in the season damage scores lower later presumably because healing had occurred. While these studies suggest a positive outlook for bats that survive an infection with WNS, it is important to remember that the conclusions were drawn from recapture data. Fuller et al. (Fuller et al.,2011) reported a 10% recapture rate but recognized that the fate of bats with wing damage that were not recaptured was unknown (see also Francl et al., 2011; Powers et al., 2013). This leaves open the possibility that bats with severe wing damage may experience limited mobility and thus die because of starvation or predation (Reichard and Kunz, 2009). In the present study, 15 of 62 (24%) *E. fuscus* we biopsied had not achieved full wound closure after one year. Additional field and lab studies are needed to assess the factors that influence how wing injuries heal to better assess the functional consequences to individuals that do not achieve full wound closure.

## MATERIALS AND METHODS

### Ethics statement

All procedures adhered to the guidelines for the care and use of wild mammals in research approved by the American Society of Mammologists (Sikes and Gannon, 2011), the Canadian Council on Animal Care, and were approved by the Animal Research Ethics Board of McMaster University.

### Animals

Wound healing studies were conducted on adult female big brown bats (*Eptesicus fuscus*) collected from the wild in southern Ontario between August 2012 and May 2013. Captive bats were housed in a free-flight husbandry facility (2.5 × 1.5 × 2.3 m) at McMaster University where the colony temperature and lighting varied with ambient conditions (Faure et al., 2009), and the bats had *ad libitum* access to mealworms (*Tenebrio molitor*), water, and an outdoor flying area (2.5 × 3.8 × 3.1 m).

### Biopsy procedure

Bats in the winter and summer treatment groups were gas anesthetized for the biopsy procedure. Bats in the lactation and non-reproductive treatment groups were not anesthetized to prevent any interference with lactation. Bats were weighed and their forearm was measured prior to being placed in an anesthesia induction chamber where they breathed a 4% isofluorane:oxygen gas mixture (flow rate = 1 L/min). During membrane biopsy, bats were placed in a custom restrainer permitting access to both wings (Ceballos-Vasquez et al., 2014). One wing was extended so the fifth digit was parallel to the body and the other digits were fully extended, and tissue was excised by applying pressure with an 8-mm diameter Sklar Tru-Punch® sterile disposable punch tool (Wilmer and Barratt, 1996). Tissue was taken from the chiropatagium of both wings, between the 4^th^ and 5^th^ digits, using the joint between the metacarpal bone and the phalanges as a landmark to standardize the punch location. Week 0 was defined as the week of the biopsy procedure.

### Experimental design

The study was conducted in two parts. Part 1 assessed the effect of seasonality (i.e. winter *versus* summer temperatures) to test the hypothesis that wound healing is slower during periods of energy conservation. Part 2 assessed the effect of reproductive status (lactating *versus* non-reproductive) to test the hypothesis that wound healing is slower during periods of energy demand.

### Part I. Effect of season on wound healing

#### Winter wound healing (January 2013)

Bats were randomly assigned to the experimental biopsy (n = 12) or control treatment groups (n = 5). Experimental bats were biopsied on the left and right wings, whereas control bats were subjected to identical handling but without membrane biopsy. All bats were weighed prior to photographing their wounds. A body condition index (BCI = mass/forearm length) score was calculated weekly, using the forearm length measured at the time of anaesthesia (Jonasson and Willis, 2011), to determine if healing bats experienced faster depletion of fat reserves and to monitor to the effects of handling and disturbance during hibernation in control bats. Water drinking behaviour was recorded with an infra-red motion detection camera (8 MP Stealth Game Camera, STC-I840IRAS1) to determine if in the frequency of drinking differed between winter biopsy and control bats. Initially, we placed reflective tape on the forearm bands of bats of experimental bat to identify individuals that were drinking; however, some bats removed the tape, so we bleached marked the fur on the back to differentiate bats belonging to the experimental and control groups (Silva et al., 2007). Videos of drinking behaviour were scored by two individuals who were blind to the assignment of bats within each treatment group.

#### Summer wound healing (July 2013)

Wing biopsies were performed on 14 adult non-reproductive female *E. fuscus* using the same procedures and biopsy location as previously described.

### Part II. Effect of reproductive status on wound healing (June 2013)

In June 2013, wing biopsies were performed on 20 lactating (experimental treatment group) and 16 non-reproductive adult female *E. fuscus* (control treatment group) using the same procedures and biopsy location as previously described. Bats were randomly chosen from the husbandry facility until the appropriate number was assigned to each treatment group. Lactating females were biopsied when their offspring were between 2 and 4 days old to ensure that pups and mothers had bonded and established a feeding routine. We also weighed and measured the forearm length of the pups to determine if wound healing by the mother influenced pup growth.

### Wound imaging and measurements

Wound healing was monitored using a procedure similar to that of Faure et al. (Faure et al., 2009). Once a week, bats were placed in a custom restrainer (Ceballos-Vasquez et al., 2014) and their wounds were photographed with a DP25 CCD camera (5 MPixel resolution; Olympus, Tokyo, Japan) mounted on an Olympus SZX10 stereomicroscope. Wound areas were measured (mm^2^) with ImageJ software (National Institute of Health), and calculated as the average area (mm^2^) for the left and right wings (Wallenstein and Brem, 2004). The proportion of wound area that had healed was calculated using a modified version of the equation by Baker et al. (Baker et al., 1997): proportion healed  =  ([initial wound area–current wound area]/initial wound area). Theoretically, the area of an 8-mm diameter circle (area = π·r^2^) is 50.27 mm^2^; however, due to overstretching of the wing membrane and human measurement error, the mean ± standard deviation initial wound area we measured was slightly larger at 52.38 ± 2.74 mm^2^ (n = 62). In four bats we measured the linear distance between the 4^th^ digit and a major blood vessel below the wound to determine the amount of tissue contraction around the biopsy area.

### Statistical analysis

Data were analysed using the SPSS statistical software package for Windows (version 21, SPSS Inc, Chicago). Unless stated otherwise, all data are reported as the mean ± standard error (SE). Data were first tested for normality (Shapiro and Wilk, 1965) and equality of variances (Bartlett *F*-test), and non-normal (heteroscedastic) data were subsequently analyzed with an equivalent nonparametric test. The BCI data collected in Part 1 of the study were not normally distributed, hence a generalized estimated equation was used to compare scores between groups. We compared the frequency of winter water drinking behaviour between experimental and control bats, including other bats living in the colony, using a Pearson chi square test. A Kaplan-Meier survival analysis was used to compare healing times between treatment groups. We report the time to complete (100%) healing as the number of weeks post biopsy when both wings no longer had open wounds when viewed under a stereomicroscope. Bats that did not completely heal by week 13 were eliminated from the analysis. In Part II two bats were removed (P14 control; Blu041 lactating) from the analysis because they had not healed to 50% by week 13.

In Part II the mass and forearm length of pups in the study of reproductive effects were used to measure growth rate across the first four weeks post-biopsy of the lactating females. Growth rate was calculated as the slope of the tangent line using the mass (or forearm length) plotted against time (weeks). A linear mixed model (R Development Core Team 2012, version 2.15.1) was used to evaluate the influence of number of offspring sired and the growth rate of the pups on the mother's healing time.

### List of abbreviations

BCI, Body Condition Index; SD, Standard deviation; SE, Standard error of the mean; WNS, White-nose syndrome.
